# Impact of Myocardial Energy Expenditure and Diastolic Dysfunction on One Year Outcome Patients With HFpEF

**DOI:** 10.3389/fphys.2022.655827

**Published:** 2022-04-04

**Authors:** Yu Wang, Yalan Cao, Shuting Xiang, Shunji Liang, Xiumei Yang, Ning Zhu, Weiyi Fang, Qin Yu

**Affiliations:** ^1^ Department of Cardiology, Affiliated Zhongshan Hospital of Dalian University, Dalian, China; ^2^ Department of Nephrology, Affiliated Xinhua Hospital of Dalian University, Dalian, China; ^3^ Dalian Medical University, Dalian, China; ^4^ Zunyi Medical University, Zunyi, China; ^5^ Department of Echocardiogram, Affiliated Zhongshan Hospital of Dalian University, Dalian, China; ^6^ Department of Cardiology, The Second Affiliated Hospital of Dalian Medical University, Dalian, China; ^7^ Department of Cardiology, Shanghai Chest Hospital, Shanghai, China

**Keywords:** HFpEF, energy metabolism, diastolic dysfunction, cardiology, MEE

## Abstract

**Objective:** To explore the correlation between characteristics of myocardial energy expenditure (MEE) and the degree of diastolic dysfunction in patients of heart failure with preserved ejection fraction (HFpEF) and its clinical significance.

**Methods:** 125 consecutive patients diagnosed with HFpEF in the Department of Cardiology, Affiliated Zhongshan Hospital of Dalian University from January 2018 to October 2018 were enrolled. According to the degree of diastolic dysfunction, patients were divided into group A (8 ≤ E/e' ≤15) and group B (E/e'> 15), and MEE was calculated, patients finished 1-year clinical follow-up.

**Results:** The level of MEE in group A was significantly lower than that in group B (*p* < 0.05). During 1-year follow up, MEE over 3145.69 kcal/systole was associated with increased risk of death as compared to patients with MEE less 3145.69 kcal/systole, and in patients with MEE over 101.68 kcal/min than in patients with MEE less than 101.68 kcal/min.

**Conclusion:** There is a significant correlation between MEE and diastolic dysfunction and MEE over 3145.69 kcal/systole as well as MEE over 101.68 kcal/min are linked with increased risk of 1-year mortality in HFpEF.

## Introduction

Heart failure (HF) is a severe manifestation of various heart diseases, associated with high rate of mortality and re-hospitalization. The worldwide prevalence of HF is 1.0–2.0%, ≥10% among people with age over 70ys is (Kuschyk et al., 2018). Heart failure with preserved ejection fraction (HFpEF) has focus on increasing attention worldwide, which accounts for around half of hospitalized patients with HF and the clinical features may be distinct from those with Heart failure with reduced ejection fraction (HFrEF). Further efforts are needed to better characterize HFpEF patients. Increased myocardial stiffness induced diastolic function-related abnormalities is generally considered to be the principal pathophysiological mechanism of HFpEF, in addition, following factors are found to contribute to the pathogenesis of HFpEF: left atrial enlargement and pulmonary hypertension, plasma volume expansion, systemic microvascular inflammation, cardiometabolic functional abnormalities, and cellular (titin)/extracellular (fibrosis) structural abnormalities (Lam et al., 2018). Recent studies have implied that myocardial energy expenditure (MEE) abnormality might also be an important determinant of HF, which serves not only a cause but also an aggravated factor of HF (McKirnan et al., 2019). Several clinical studies defined the role of MEE in HFrEF patients and its clinical significance (Mann, 2017; Mehmet et al., 2017; Bhatt and Butler, 2018), but the present study aimed to explore the myocardial energy consumption in patients with HFpEF. Cardiac magnetic resonance wave method, metabolomics and noninvasive echocardiography could be used to define MEE. Echocardiography could reveal subclinical HF and predict risk of subsequent events and tension time index (TTI) has been recognized as the most accurate and direct myocardial oxygen consumption index via non-invasive echocardiogram, that is MEE (Sarnoff et al., 1958; Shimizu et al., 1991). As a widely applicable measure, echocardiography is used to evaluate myocardial mechanics. Echocardiography-based data and calculation of MEE are both available and feasible. The present study aimed to investigate the correlation between MEE and the diastolic dysfunction and its clinical significance in HFpEF patients.

### Study Population

Patients who were admitted to the Department of Cardiology of Affiliated Zhongshan Hospital of Dalian University and diagnosed as HFpEF from January 2018 to October 2018 were selected to participate in the study. The study protocol was approved and carried out in accordance with recommendations of the ethic committee of Affiliated Zhongshan Hospital of Dalian University. The total of 125 of enrolled participates have signed the informed consents in accordance with the Declaration of Helsinki (World Medical Association, 2013). They were divided into group A (8 ≤ E/e'≤15, *n* = 69, aged 70.90 ± 10.97 years, 46.4% males) and group B (E/e' >15, *n* = 56, aged 71.98 ± 10.39 years, 50% males).

The peak blood flow (E) at the early stage of mitral valve diastole was measured according to the color Doppler echocardiogram. Left ventricular ejection fraction (LVEF) was measured at 1 year follow-up. The primary endpoint is the all-cause mortality at the 1-year follow-up.

### Inclusion Criteria

HFpEF was defined according to 2016 ESC Guidelines for the diagnosis and treatment of acute and chronic HF (Ponikowski et al., 2016): 1) HF symptoms and/or signs; 2) LVEF≥50%; 3) Elevated levels of natriuretic peptides and at least one additional criterion: a. Left ventricular hypertrophy and left atrial enlargement b. abnormal diastolic function. We defined the diastolic dysfunction as follows: Patients met one of the following echocardiographic criteria were defined as diastolic dysfunction: LAVI >34 ml/m^2^, septal E/e’ ratio>15, and TRVmax>2.8 m/s (McMurray et al., 2012; Nagueh et al., 2016).

### Exclusion Criteria

1) Unable to express medical history and co-examination due to unconsciousness, mental retardation or vague speech; 2) Combined with malignant hypertension, acute myocardial infarction, aortic dissection, severe arrhythmia (ventricular tachycardia, atrial fibrillation, atrial flutter, severe tachyarrhythmia or bradycardia) or pacemaker implant; 3) Various congenital heart diseases; 4) Heart valve disease, pericardial disease, hypertrophic cardiomyopathy; 5) Take medicine that improves myocardial energy metabolism in the past 3 months (trimetazidine, coenzyme Q10); 6) Those who have serious diseases of other systems, such as malignant tumors, liver cirrhosis, renal failure, and bleeding diseases.

### Research Method

Patient’s morning height (H): The patient stood upright on the floor of the height meter on bare foot, with the heel, metatarsal bone, and the midpoint of shoulder blades abutting on the height meter post. The level plate of the altimeter was moved to the top of the patient’s head. The recorder’s line of sight was flushed with the lowest point of the upper edge of the patient’s tragus and the lower edge of the orbit. Measurement was recorded the value in meters.

Morning weight (W): The patient took off clothes and socks, stood on the weight scale, and the value was recorded in kilograms.

Laboratory parameters, including blood lipid, fast blood glucose, glycated hemoglobin and N-terminal pro-brain natriuretic peptide (NT-proBNP) were obtained in all participants. Tandem mass spectrometry was used to detect valine, leucine, and free carnitine with peripheral blood collected by dried blood spot method. Blood pressure, heart rate, electrocardiogram and 6-min walking test (6MWT) were measured after hospital admission.

All the echocardiographic parameters including left ventricular ejection fraction (LVEF), peak blood flow (E) in early mitral valve diastole, early mitral annulus velocity (e'), stroke volume (SV), tricuspid regurgitation velocity (TR velocity), ventricular septal thickness (IVSD), left ventricular posterior wall systolic thickness (PWTs) and diastolic thickness (PWTd), left ventricular end-systolic diameter (LVIDs), end-diastolic diameter (LVIDd) were measured with PHILIPS EPIQ7 ultrasound system, E/e' as well as left ventricular short axis shortening rate (FS%) was calculated. Aortic valve ejection time (ET) was measured by Doppler blood flow spectrum in three continuous cardiac cycles, and the average value was obtained. The mean arterial pressure (MAP), cardiac output (CO), cardiac index (CI), left atrial volume index (LAVI), left ventricular mass index (LVMI), left ventricular end-systolic peripheral wall stress (cESS) were calculated, and MEE was calculated by related formula (Devereux et al., 1986; Palmieri et al., 2003; Dokainish et al., 2004; Paulus et al., 2007; Palmieri et al., 2008; Galderisi, 2011). A skilled and experienced echocardiography doctor was appointed to perform the examination on the whole participants in this study to ensure the quality of the examination.

BSA=0.006×H+0.0128×W−0.153

$$\text{L}\text{V}\text{M}\text{I}=\frac{\text{0}\text{.}8\text{×}1\text{.}04\text{ × }\left[\;{(\text{LVIDd }+\text{IVSD}+\text{PWTd}\;)}^{3}{-\text{LVIDd}}^{3}\right]+\text{0}\text{.}6}{\text{BSA}}$$

$$\text{c}\text{E}\text{S}\text{S}=\frac{\text{SBP}\times {\left(\text{LVIDS/}2\right)}^{2}\times \left\{1+\frac{{\left(\text{LVIDs/}2\;+\;\text{PWTs}\right)}^{2}}{{\left(\text{LVIDs/}2\;+\;\text{PWTs/}2\right)}^{2}}\right\}}{{\left(\text{LVIDs/}2+\text{PWTs}\right)}^{2}{-\left(\text{LVIDs/}2\right)}^{2}}$$

MEE (kcal/min) = MEE (kcal/systole) × HR = cESS × LVET × LVSV × HR × 4.2 × 10^–4^

### Statistical Analysis

The continuous variables were reported as the mean ± standard deviation (SD) and the categorical variables were expressed as the number of patients and percentages. Kolmogorov-Smirnov tests were used to assess the normality of the data distribution. The comparisons between groups were performed with *t* test (for continuous variables), Mann-Whitney *U* test (for non-parametric variables). Spearman’s correlation analysis. Receiver operating characteristics (ROC) curve analysis was carried out to investigate the efficacy of MEE and Metabolomics in predicting end point and the best cut-off values were determined on the basis of Youden index. Based on these cut-off values, patients were divided into two groups for each parameter. Kaplan–Meier curve analysis was used for event-free survival analysis in between these groups. Statistical significance was defined as *p* < 0.05. Data was analyzed with SPSS 25.0 software.

## Results

The clinical data of the whole participates was shown in Table 1. It was shown as both group A (8 ≤ E/e'≤15) and group B (E/e' >15), The two groups were matched with demographic, clinical and data of laboratory examination (*p* > 0.05).

**TABLE 1:** The baseline characteristics and laboratory parameters of study population.

Variables	A	B	*p*
Age(y)	70.90 ± 10.97	71.98 ± 10.39	0.570
Male gender, n (%)	32(46%)	28(50%)	0.687
Hypertension, n (%)	25(36%)	35(63%)	0.820
Diabetes mellitus, n (%)	14(20%)	11(20%)	0.441
Coronary heart disease, n (%)	9(13%)	15(27%)	0.290
BMI (kg/m^2^)	25.60 ± 4.23	25.74 ± 3.69	0.945
BSA (m^2^)	1.73 ± 0.19	1.74 ± 0.19	0.941
MAP (mmHg)	95.27 ± 9.84	99.01 ± 12.41	0.197
HR (time/min)	75.77 ± 15.06	74.07 ± 14.30	0.471
6MWT(M)	426.09 ± 163.68	373.66 ± 137.34	0.054
TG (mmol/L)	1.53 ± 1.28	1.59 ± 1.13	0.650
CHO (mmol/L)	4.70 ± 1.80	4.40 ± 1.36	0.276
HDL-C (mmol/l)	1.27 ± 0.52	1.15 ± 0.35	0.144
LDL-C (mmol/l)	2.61 ± 1.02	2.51 ± 0.94	0.439
FBG (mmol/L)	6.78 ± 3.01	6.73 ± 3.04	0.984
GHb(%)	6.70 ± 1.91	6.60 ± 1.46	0.901
NT-proBNP(pg/ml)	889.92 ± 651.19	903.21 ± 668.61	0.197
Antiplatelet, n (%)	27(39%)	22(39%)	0.800
β-blockers, n (%)	24(35%)	18(32%)	0.702
Statins, n (%)	41(59%)	36(64%)	0.256
CCB, n (%)	28 (41%)	20 (36%)	0.301
ACEI/ARB, n (%)	32 (46%)	36 (61%)	0.069
Valine (umol/l)	128.09 ± 29.23	125.59 ± 31.56	0.492
Leucine (umol/l)	105.76 ± 33.78	103.45 ± 31.51	0.768
Free carnitine (umol/l)	41.00 ± 35.74	37.48 ± 31.03	0.335

Data were shown as Mean ± Standard Deviation, Group A: 8 ≤ E/e'≤15 (*n* = 69), Group B: E/e'>15 (*n* = 56), Statistical significance was defined as *p* < 0.05, BMI, body mass index; BSA, body surface area; MAP, mean arterial pressure; HR, heart rate; 6MWT, 6 min walk test; TG, triglyceride; CHO, cholesterol; HDL-C, high density lipoprotein cholesterol; LDL-C, low density lipoprotein cholesterol; FBG, fast blood glucose; NT-proBNP, N-terminal pro-brain natriuretic peptide; CCB, calcium channel blocker; ACEI, angiotensin converting enzyme inhibitors; ARB, angiotensin receptor blocker.

**FIGURE 1:**
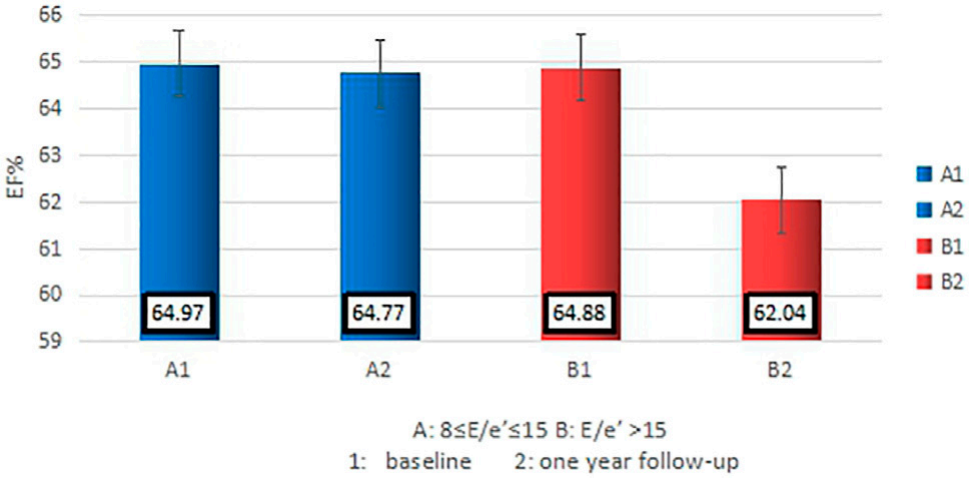
The basiline and the 1 year follow-up LVEF of participants.

**FIGURE 2:**
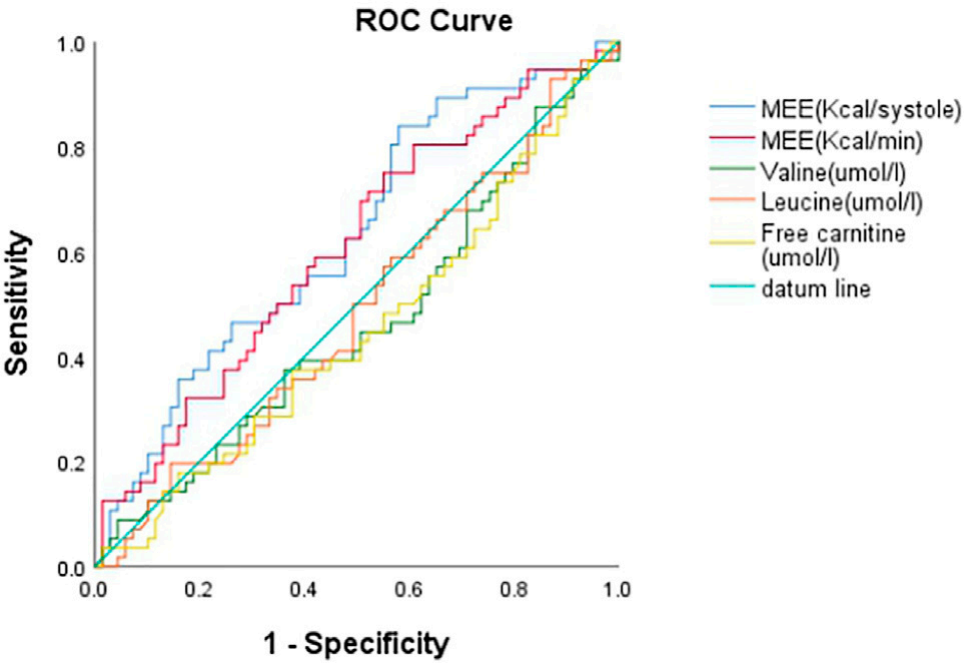
ROC curve of MEE (cal/systole), MEE (Kcal/min), Valine (umol/l), Leucine (umol/l) and Free carnitine (umol/l).

**FIGURE 3:**
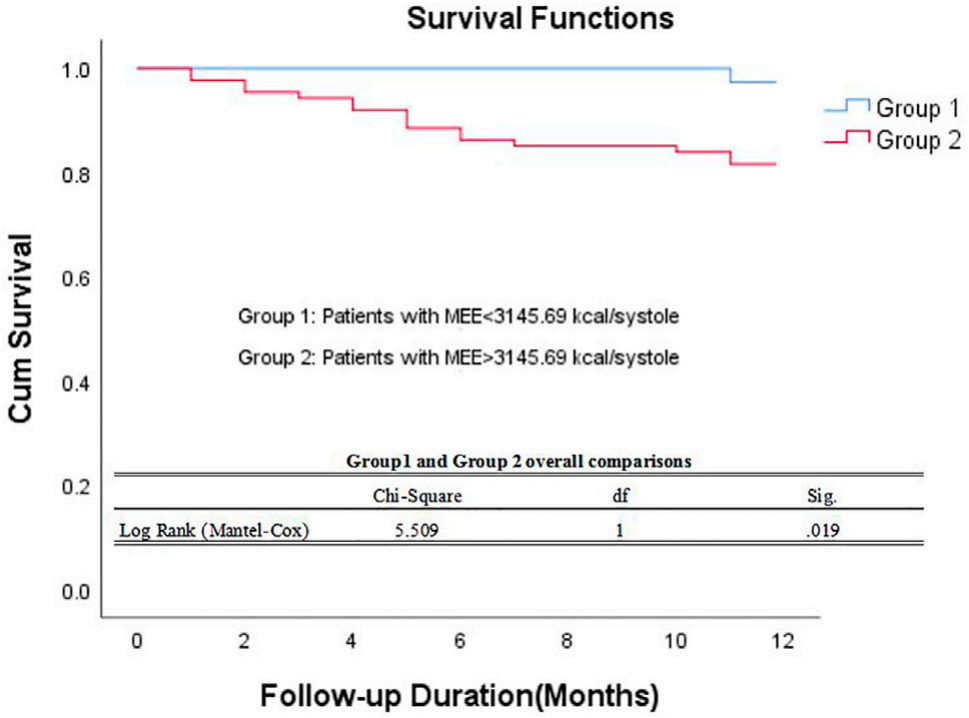
Kaplan–Meier survival curve of participants. Demonstrating long-term all-cause mortality among patients groups specified based on myocardial energy expenditure cut-off value 3145.69 kcal/systole. MEE myocardial energy expenditure.

**FIGURE 4:**
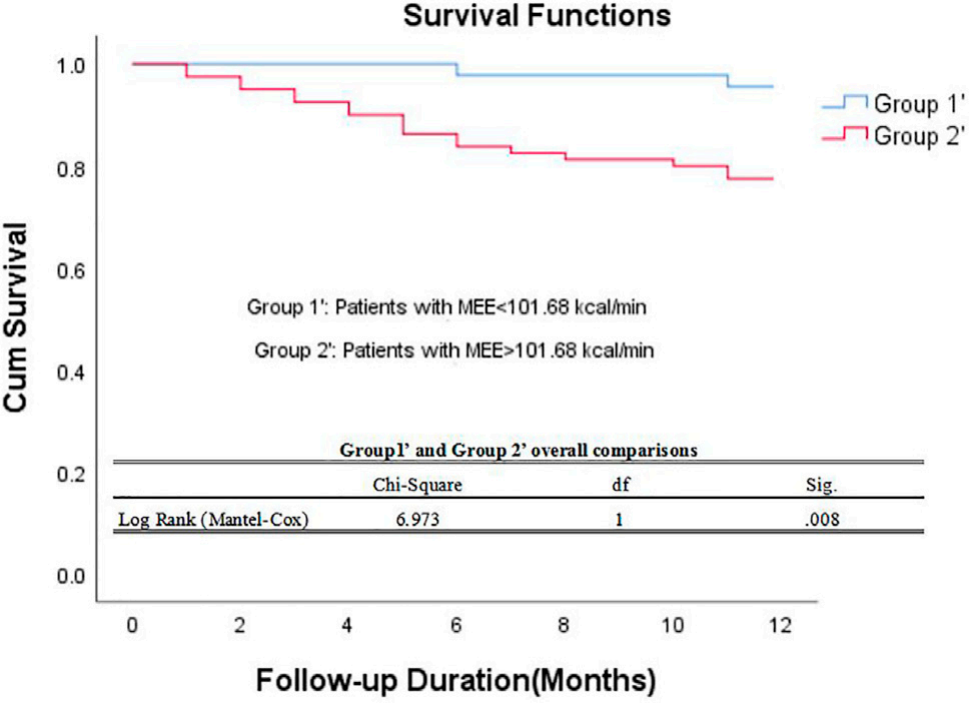
Kaplan–Meier survival curve of participants. Demonstrating long-term all-cause mortality among patients groups specified based on myocardial energy expenditure cut-off value 101.68 kcal/min. MEE myocardial energy expenditure.

The data of echocardiography parameters and MEE was presented in Table 2. The MEE per systole (3944.74 ± 2119.06 vs. 4611.67 ± 1875.28 kcal, *p* = 0.013) and MEE per minute (123.01 ± 65.47 vs. 144.83 ± 70.80 kcal, *p* = 0.040) were lower in group A than in group B; MEE was positively correlated with NT-proBNP (*r* = 0.202, *p* = 0.027) and negatively correlated with the 6-min walking distance (*r* = −0.305, *p* = 0.001) (Table 3). However, there was no difference in sex and age between the two groups (*p* > 0.05). MEE per minute in overweight (BMI≥24) group was higher than the other group (142.01 ± 76.37 vs.59.99 ± 6.95/116.00 ± 41.37 kcal, *p* < 0.05), MEE per minute was lower in mild degree HF group (6MWT>450) than in moderate degree HF group (150<6MWT≤450) (106.61 ± 46.33 vs.149.03 ± 72.51 kcal, *p* < 0.05) (Table 4); During 1 year follow-up, LVEF was higher in group A than in group B (64.77% ± 4.74 vs. 62.04% ± 6.39, *p* = 0.033). Additionally, LVEF were lower than baseline in both two groups (64.97% ± 4.57 vs. 64.77% ± 4.74, *p* < 0.05) and (64.88% ± 4.59 vs. 62.04% ± 6.39, *p* < 0.05) (Table 2; Figure 1); In ROC analysis, MEE (kcal/systole) cut-off value of 3145.69 had 84% sensitivity and 42% specificity for prediction of composite end-point (AUC = 0.63, *p* = 0.013) (Table 5; Figure 2). All the subjects were divided into group 1 and 2 again based on the MEE cut off value 3145.69 (Group 1: 38 patients, MEE <3145.69 kcal/systole; Group 2: 87 patients, MEE >3145.69 kcal/systole). Kaplan-Meier analysis according to the long-term event-free survival revealed that occurrence of events was lower in Group 1 compared to Group 2 (*p* = 0.019) (Figure 3); MEE (kcal/min) cut-off value of 101.68 has 75% sensitivity and 45% specificity for prediction of composite end-point (AUC = 0.61, *p* = 0.040) (Table 5; Figure 2). The study population was divided into Group 1’ and Group 2’ based on the MEE cut off value 101.68 (Group 1’: 45 patients, MEE <101.68 kcal/min; Group 2’: 80 patients, MEE >101.68 kcal/min). Kaplan-Meier analysis according to the long-term event-free survival revealed the lower occurrence of events in Group 1’ compared with Group 2’ (*p* = 0.008) (Figure 4).

**TABLE 2:** The baseline Echocardiogram measurements of study population.

Variables	A	B	*p*
CO(L/min)	4.43 ± 1.72	4.30 ± 1.55	0.691
CI(L/min*m^2^)	2.50 ± 0.88	2.46 ± 0.91	0.664
LVEF (%)-baseline	64.97 ± 4.57	64.88 ± 4.59	0.746
-1-year follow-up	64.77 ± 4.74^△^	62.04 ± 6.39^△^	0.033
SV(ML)	74.52 ± 16.04	73.27 ± 16.85	0.769
TR velocity (m/s)	2.47 ± 0.63	2.64 ± 0.63	0.098
LVFS (%)	35.45 ± 5.45	34.50 ± 4.26	0.514
LAVI (ml/m^2^)	34.37 ± 5.69	36.24 ± 6.96	0.078
LVMI(g/m^2^)	121.16 ± 34.78(M)110.56 ± 22.92(F)	123.34 ± 42.51(M)130.18 ± 41.57(F)	0.894(M)0.079(F)
E/e' ratio	12.07 ± 2.31	21.75 ± 7.59	0.000
cESS(Kdyne/cm^2^)	171.62 ± 74.79	203.50 ± 72.68	0.002
MEE(Kcal/systole)	3944.74 ± 2119.06	4611.67 ± 1875.28	0.013
MEE(Kcal/min)	123.01 ± 65.47	144.83 ± 70.80	0.040

Data were shown as Mean ± Standard Deviation, Group A: 8 ≤ E/e'≤15 (*n* = 69), Group B: E/e'>15 (*n* = 56), Statistical significance was defined as *p* < 0.05, ^△^ *p* < 0.05 vs. Baseline, CO, cardiac output; CI, cardiac index; LVEF, left ventricular ejection fraction; SV, stroke volume; TR, tricuspid valve; LVFS, left ventricular fraction shortening; LAVI, left atrial volume index; LVMI, left ventricular mass index; E/e', peak blood flow (E) in early mitral valve diastole/early mitral annulus velocity (e'); cESS, circumferential end-systolic wall stress; MEE, myocardial energy expenditure.

**TABLE 3:** The correlation between MEE and multiple variables.

Variables	r	*p*
E/E′	0.145	0.107
6MWT(m)	−0.305	0.001
NT-proBNP(pg/ml)	0.202	0.027
LVMI(g/m^2^)	0.091	0.325
LVEF (%)	−0.171	0.057

Statistical significance was defined as *p* < 0.05, MEE, myocardial energy expenditure, 6MWT, 6-min walking test; LVMI, left ventricular mass index; LVEF, left ventricular ejection fraction.

**TABLE 4:** Subgroups of MEE.

Variables		MEE (kcal/min)
Gender,n (%)	Male (48%)	126.53 ± 68.01
	Female (52%)	138.57 ± 68.97
Age,n (%)	≤65 (33%)	137.56 ± 89.03
	66∼79 (38%)	123.66 ± 48.52
	≥80 (29%)	139.98 ± 65.31
BMI,n (%)	<18.5 (2%)	59.99 ± 6.95
	18.5∼24 (30%)	116.00 ± 41.37^△^
	≥24 (68%)	142.01 ± 76.37^△*^
6MWT, n (%)	≤150 (4%)	150.28 ± 116.46
	150∼450 (58%)	149.03 ± 72.51
	>450 (32%)	106.61 ± 46.33^#^

Data were shown as Mean ± Standard Deviation, statistical significance was defined as *p* < 0.05, ^△^ *p* < 0.05 vs. BMI<18.5, *p < 0.05 vs. 18.5 ≤ BMI<24, ^#^ *p* < 0.05 vs. 150<6MWT≤450, MEE, myocardial energy expenditure; BMI, body mass index, 6MWT, 6-min walking test.

**TABLE 5:** The comparison of area under ROC curve of MEE and metabolomics.

	AUC	*p* Value	Cutoff	Sensitivity (%)	Specificity (%)	SD	95% CI
MEE (Kcal/systole)	0.63	0.013	3145.69	84	42	0.05	(0.53, 0.73)
MEE (Kcal/min)	0.61	0.040	101.68	75	45	0.05	(0.51, 0.71)
Valine (umol/l)	0.46	0.492	178.56	9	96	0.05	(0.36, 0.57)
Leucine (umol/l)	0.49	0.768	68.65	93	13	0.05	(0.38, 0.59)
Free carnitine (umol/l)	0.45	0.335	19.46	96	6	0.05	(0.35, 0.55)

MEE, myocardial energy expenditure; AUC, area under the curve; SD, standard deviation; CI, confidence interval.

## Discussion

Data released in 2012 shows that the prevalence of HF in developed countries will increase by 46% by 2030, and the incidence of HF will double for every 10-year increased in age (Roger et al., 2012). Several previous studies have shown that the HFpEF patients account for half or more of the total HF patients, and their mortality are no less than those with HFrEF (Campbell et al., 2012). Cardiomyocyte necrosis and fibrosis might also contribute to insufficient energy supply and utilization disorder, which could be another important pathomechanism of HF. The disturbance on myocardial energy metabolism might also be an important determinant responsible for the continuous progression of HFpEF, Detection and monitoring of MEE might thus be of particularly importance in the early screening for HFpEF patients with increased risk of poor prognosis.

Some experts pointed out that MEE increased significantly with the decrease of LVEF and the increase of cardiac function grade, and was closely related to the NT-proBNP level (Cetin et al., 2018; Chen et al., 2019a). It is necessary to focus on cardiac energy metabolism in patients with HFpEF. Some non-invasively Techniques including PET, MRI and echocardiography have been used to assess MEE, Echocardiography is specially easy available and well follow-up instrument for calculation of MEE. In our study, we have inserted the function formula and algorithm software into the echocardiography device to simplify the computation.

Our preliminary study findings demonstrated that E/e' ratio, cESS (Kdyne/cm^2^), MEE (Kcal/systole), MEE (Kcal/min) in E/e' >15 group is significantly higher than that in 8 ≤ E/e'≤15 group. LAVI values and TR velocity in E/e' >15 group had a tendency of higher than that in 8 ≤ E/e'≤15 group. For HFpEF patients, MEE is significantly correlation with the degree of diastolic dysfunction. The results suggested that the worse the diastolic function of HFpEF, the more energy consumption per minute and even per stroke is, In other words, the augmented myocardial oxygen and energy consumption, together with aggravated neurohumoral activity might coexist in HFpEF patients with severely diastolic dysfunction, which may further lead to the depletion of cardiac energy reserves in HFpEF patients and establish the vicious circle of above unfavorable features in HFpEF patients.

After 1 year follow-up, Both 8 ≤ E/e'≤15 and E/e' >15 patients had lower LVEF than that in baseline. Which indicated a continuous deleterious nature of HFpEF. In the early stage of HF, the metabolism of myocardial energy substrate is nearly normal, while in the advanced stage, the utilization of fatty acid to oxidize myocardium decreased and the utilization of glucose changed. In other words, in the early stage of HFpEF, the metabolism of myocardial energy substrate is probably normal, but in advanced stage, with increased myocardial oxygen and energy consumption, abnormal MEE might reflect the progressive systolic deterioration in HFpEF patients (Taegtmeyer et al., 2016; Fillmore et al., 2018).

Heart is an organ with high activity and energy consumption. The heart muscle movement, either relaxes or contracts, needs sufficient energy supply. The MEE in this study was negatively correlated with the 6-min walking distance and positively correlated with NT-proBNP. MEE reflects the severity of heart failure in patients with ejection fraction-preserved heart failure. This study expands the evidence that accurate assessment of MEE is helpful to reflect the severity of HF and guide clinical medication. (Valvular heart disease will interfere with the volume loading and hemodynamic factors, so we excluded these patients, and renal failure patients due to the influence of cardiorenal syndrome, BNP is not accurate).

In the study of Chen (Chen et al., 2019), branched amino acids (valine, leucine, isoleucine) are abnormal amino acids, and their increases can increase the risk of cardiovascular disease, while free carnitine is an essential substance for fatty acid metabolism. Any activity that leads to abnormal energy metabolism can lead to cardiac mechanical systolic and diastolic dysfunction, and then induce ventricular remodeling. When heart energy metabolism is abnormal, heart function will deteriorate (Guzun et al., 2011). In our study, 8 ≤ E/e'≤15 patients had a tendency of higher values (valine, leucine, free carnitine) than E/e' >15 group. Diastolic patients in the gray area produce sufficient amounts of ATP by using their enhanced metabolism. From a different point of view, a boosted energetic metabolism in patients with 8 ≤ E/e'≤15 resulted in a depletion of metabolic capacity of myocardium leading to a progressive deterioration in more advanced patients with HfpEF. On the grounds, these results suggest that patients who diastolic dysfunction in gray area (8 ≤ E/e'≤15) need more attention.

However, We found that compared with metabolomics, the method of MEE measured by echocardiogram could reflect the degree of diastolic dysfunction of patients with HFpEF better, and also has predictive value. What’s more, this study reflect that patients with HFpEF who are elderly, overweight, female sex population spend more MEE, suggesting that elderly, obese and female patients should undergo more detailed screening, to improve the detection rate of HFpEF. This conclusion same as found that women, African American, obese, and hypertensive have more HFpEF patients, and upregulated genes in oxidative phosphorylation pathways in HFpEF were associated with obesity (Choi et al., 2018; Hahn et al., 2020).

Although some drugs known to have remodeling effect in the myocardial tissue (Toh et al., 2016) which could increase glucose oxidation rate and reduce myocardial oxygen consumption delay HFpEF progression. Calcium channel blockers reduce the intracellular calcium concentration and improve the active diastolic function of cardiomyocytes. The patients enrolled received various medication shown Table 1 is insufficient to explore the impact of various drug used on diastolic function profile in this study. Future studies are warranted to address this issue.

The worse the diastolic function, the greater the myocardial energy consumption to maintain the heart function to some extent. HFpEF trials like VICTORIA (Armstrong et al., 2020), EMPEROR-Preserved (Baseline Characteristics, 2016), PARAGON-HF (Ferrari et al., 2020), TOPCAT (McMurray and O'Connor, 2014), I-PRESERVE (McMurray et al., 2014), CHARM-Preserved (Hermann et al., 2004) often did not measure MEE and further clinical studies are wanted to validate if adding MEE as measurement index could match the observed clinical changes induced by the intervention. What’s more, we will extend the follow-up time and collect treatment options for patients includes the medication status of the underlying disease to provide effective information for further future follow-up. Recently hospitalized HFpEF patients have a two to three fold increased risk of cardiovascular death. It is same as our 1 year follow-up data. In ROC analysis, MEE displayed a higher AUC than other parameters. Therefore, we postulate that MEE might be a valuable predictor of prognosis in HFpEF patients.

The latest study defined Diastolic dysfunction (DD) severity with three predominant parameters (i.e. LAVi, septal E/E′, and TRVmax) is for and predicting the outcome in HFmrEF and HFrEF patients with various cardiac rhythms. Severe DD serves as an independent determinant for all-cause mortality for both HFmrEF and HFrEF patients after adjustment of clinical and other echocardiographic covariates. This finding might be interpreted as a hint for DD being the ‘straw that breaks the camel’s back’ for CV death in HFrEF, but not in HFmrEF patients. MEE might be a part of mechanism (Liu et al., 2021). As a simple, feasible and noninvasive way to evaluate MEE, we showed that echocardiogram-derived MEE correlates well the severity of diastolic dysfunction in patients with HFpEF. MEE might thus be used as a clinical parameter to monitor the HFpEF patients and serves as a biomarker of risk stratification for the prognosis of HFpEF.

## Limitations

The deficiency of this study is that the 1 year follow up data is limited to LVEF and mortality, without detailed diastolic function MEE calculations, and It is to note that the number of enrolled patients is relatively small and from a single medical center in our study, and future studies with large patient cohort are needed to validate present findings. Patients were followed up for a long time to observe the clinical symptoms, cardiac function classification, objective biochemical, echocardiogram indicators and quality of life. In this study all-cause mortality was analyzed during follow-up and cardiovascular mortality, MACE and HF rehospitalization data were not collected during follow-up, future studies is required to overcome this study limitation.

It is of importance to observe the serial changes of MEE parameters and explore the impact dynamic MEE changes on outcome of HFpEF patients and observe the impact of medication on these parameters and the dynamic prognostic implication of theses parameters, the upcoming study in our center will address this issue.

## Data Availability

The original contributions presented in the study are included in the article/Supplementary Material, further inquiries can be directed to the corresponding author.

## References

[B1] Armstrong P. W. Pieske B. Anstrom K. J. Ezekowitz J. Hernandez A. F. Butler J. (2020). Vericiguat in Patients with Heart Failure and Reduced Ejection Fraction. New Engl. J. Med. 20, 382. 10.1056/NEJMoa1915928 32222134

[B2] Baseline Characteristics of Patients with Heart Failure with Preserved Ejection Fraction in the EMPEROR-Preserved Trial[J]. 2016. 80. Eur. J. Heart Fail.. 10.1002/ejhf.2064 33251670

[B3] Bhatt K. N. Butler J. (2018). Myocardial Energetics and Heart Failure: a Review of Recent Therapeutic Trials[J]. Curr. Heart Fail. Rep. 15 (10), 191–197. 10.1007/s11897-018-0386-8 29707741

[B4] Campbell R. T. Jhund P. S. Castagno D. Hawkins N. M. Petrie M. C. McMurray J. J. (2012). What Have We Learned about Patients with Heart Failure and Preserved Ejection Fraction from DIG-PEF, CHARM-Preserved, and I-PRESERVE? J. Am. Coll. Cardiol. 60 (23), 2349–2356. 10.1016/j.jacc.2012.04.064 23141494

[B5] Cetin M. S. Ozcan Cetin E. H. Canpolat U. Sasmaz H. Temizhan A. Aydogdu S. (2018). Prognostic Significance of Myocardial Energy Expenditure and Myocardial Efficiency in Patients with Heart Failure with Reduced Ejection Fraction. Int. J. Cardiovasc. Imaging 34 (2), 211–222. 10.1007/s10554-017-1226-8 28808841

[B6] Chen L. Song J. Hu S. (2019). Metabolic Remodeling of Substrate Utilization during Heart Failure Progression. Heart Fail. Rev. 24 (1), 143–154. 10.1007/s10741-018-9713-0 29789980

[B7] Chen P. Zhan Q. Bai Y. Huang X. Wang P. Pan Y. (2019). Serum Peroxisome Proliferator-Activated Receptor Gamma Coactivator-1α Related to Myocardial Energy Expenditure in Patients with Chronic Heart Failure. Am. J. Med. Sci. 357 (3), 205–212. 10.1016/j.amjms.2018.12.002 30638602

[B8] Choi K. H. Lee G. Y. Choi J. O. Jeon E. S. Lee H. Y. Cho H. J. (2018). Outcomes of De Novo and Acute Decompensated Heart Failure Patients According to Ejection Fraction. Heart 104 (6), 525–532. 10.1136/heartjnl-2017-311813 28982721

[B9] Devereux R. B. Alonso D. R. Lutas E. M. Gottlieb G. J. Campo E. Sachs I. (1986). Echocardiographic Assessment of Left Ventricular Hypertrophy: Comparison to Necropsy Findings. Am. J. Cardiol. 57 (6), 450–458. 10.1016/0002-9149(86)90771-x 2936235

[B10] Dokainish H. Zoghbi W. A. Lakkis N. M. Al-Bakshy F. Dhir M. Quinones M. A. (2004). Optimal Noninvasive Assessment of Left Ventricular Filling Pressures: a Comparison of Tissue Doppler Echocardiography and B-type Natriuretic Peptide in Patients with Pulmonary Artery Catheters. Circulation 109 (20), 2432–2439. 10.1161/01.CIR.0000127882.58426.7A 15123522

[B11] Ferrari R. Fucili A. Rapezzi C. (2020). Understanding the Results of the PARAGON-HF Trial: Viewpoint.[J]. Eur. J. Heart Fail. 22, 1522. 10.1002/ejhf.1797 32212295

[B12] Fillmore N. Levasseur J. L. Fukushima A. Wagg C. S. Wang W. Dyck J. (2018). Uncoupling of Glycolysis from Glucose Oxidation Accompanies the Development of Heart Failure with Preserved Ejection Fraction. Mol. Med. 24 (1), 3. 10.1186/s10020-018-0005-x 30134787 PMC6016884

[B13] Galderisi M. (2011). Diagnosis and Management of Left Ventricular Diastolic Dysfunction in the Hypertensive Patient. Am. J. Hypertens. 24. (5), 507–517. 10.1038/ajh.2010.235 21164497

[B14] Guzun R. Timohhina N. Tepp K. Gonzalez-Granillo M. Shevchuk I. Chekulayev V. (2011). Systems Bioenergetics of Creatine Kinase Networks: Physiological Roles of Creatine and Phosphocreatine in Regulation of Cardiac Cell Function. Amino Acids 40 (5), 1333–1348. 10.1007/s00726-011-0854-x 21390528

[B15] Hahn V. S. Knutsdottir H. Luo X. Bedi K. Margulies K. B. Haldar S. M. (2020). Myocardial Gene Expression Signatures in Human Heart Failure with Preserved Ejection Fraction. Circulation. 10.1161/CIRCULATIONAHA.120.050498 PMC7856095 33118835

[B16] Hermann F. Ruschitzka F. T. Schiffrin E. L. (2004). Clinical Trials Report. CHARM-Preserved Trial.[J]. Curr. Hypertens. Rep. 6 (1), 48., 10.1007/s11906-004-0010-z 14972093

[B17] Kuschyk J. Rudic B. Liebe V. Tülümen E. Borggrefe M. Akin I. (2018). Cardiac Contractility Modulation for Treatment of Chronic Heart Failure. Herzschrittmachertherapie & Elektrophysiologie. 29 (4), 369–376. 10.1007/s00399-018-0600-0 30361862

[B18] Lam C. S. P. Voors A. A. de Boer R. A. Solomon S. D. van Veldhuisen D. J. (2018). Heart Failure with Preserved Ejection Fraction: from Mechanisms to Therapies. Eur. Heart J. 39 (30), 2780–2792. 10.1093/eurheartj/ehy301 29905796

[B19] Liu D. Hu K. Lau K. Kiwitz T. Robitzkat K. Hammel C. (2021). Impact of Diastolic Dysfunction on Outcome in Heart Failure Patients with Mid-range or Reduced Ejection Fraction. ESC Heart Fail. 8, 2802–2815. 10.1002/ehf2.13352 33932134 PMC8318417

[B20] Mann Do. L. (2017). Targeting Myocardial Energetics in the Failing Heart: Are We There Yet?[J]. Circ. Heart Fail. 10 (12), e004658. 10.1161/CIRCHEARTFAILURE.117.004658 29217758 PMC5728660

[B21] McKirnan M. D. Ichikawa Y. Zhang Z. Zemljic-Harpf A. E. Fan S. Barupal D. K. (2019). Metabolomic Analysis of Serum and Myocardium in Compensated Heart Failure after Myocardial Infarction. Life Sci. 221, 212–223. 10.1016/j.lfs.2019.01.040 30731143 PMC6445392

[B22] McMurray J. J. Adamopoulos S. Anker S. D. Auricchio A. Böhm M. Dickstein K.;. ESC Guidelines for the Diagnosis and Treatment of Acute and Chronic Heart Failure 2012: The Task Force for the Diagnosis and Treatment of Acute and Chronic Heart Failure 2012 of the European Society of Cardiology. Developed in Collaboration with the Heart Failure Association (HFA) of the ESC. Eur Heart J. 2012 Jul;33(14):1787–1847. 10.1093/eurheartj/ehs104 22611136

[B23] McMurray J. J. V. Carson P. E. Komajda M. McKelvie R. Zile M. R. Ptaszynska A. (2014). Heart Failure with Preserved Ejection Fraction: Clinical Characteristics of 4133 Patients Enrolled in the I-PRESERVE trial.[J]. Eur. J. Heart Fail. 10 (2), 149–156. 10.1016/j.ejheart.2007.12.010 18279770

[B24] McMurray J. J. V. O'Connor C. (2014). Lessons from the TOPCAT trial.[J]. New Engl. J. Med. 370 (15), 1453–1454. 10.1056/NEJMe1401231 24716685

[B25] Mehmet S. C. (2017). Prognostic Significance of Myocardial Energy Expenditure and Myocardial Efficiency in Patients with Heart Failure with Reduced Ejection Fraction[J]. Int. J. Cardiovasc. Imaging. 10.1007/s10554-017-1226-8 28808841

[B26] Nagueh S. F. Smiseth O. A. Appleton C. P. Byrd B. F. 3rd Dokainish H. Edvardsen T. (2016). Recommendations for the Evaluation of Left Ventricular Diastolic Function by Echocardiography: An Update from the American Society of Echocardiography and the European Association of Cardiovascular Imaging. J. Am. Soc. Echocardiogr. 29, 277–314. 10.1016/j.echo.2016.01.011.PMID:27037982 27037982

[B27] Palmieri V. Bella J. N. Arnett D. K. Oberman A. Kitzman D. W. Hopkins P. N. (2003). Associations of Aortic and Mitral Regurgitation with Body Composition and Myocardial Energy Expenditure in Adults with Hypertension: the Hypertension Genetic Epidemiology Network Study. Am. Heart J. 145 (6), 1071–1077. 10.1016/S0002-8703(03)00099-1 12796765

[B28] Palmieri V. Roman M. J. Bella J. N. Liu J. E. Best L. G. Lee E. T. (2008). Prognostic Implications of Relations of Left Ventricular Systolic Dysfunction with Body Composition and Myocardial Energy Expenditure: the Strong Heart Study. J. Am. Soc. Echocardiography 21 (1), 66–71. 10.1016/j.echo.2007.05.008 PMC4294423 17628407

[B29] Paulus W. J. Tschöpe C. Sanderson J. E. Rusconi C. Flachskampf F. A. Rademakers F. E. (2007). How to Diagnose Diastolic Heart Failure: a Consensus Statement on the Diagnosis of Heart Failure with normal Left Ventricular Ejection Fraction by the Heart Failure and Echocardiography Associations of the European Society of Cardiology. Eur. Heart J. 28 (20), 2539–2550. 10.1093/eurheartj/ehm037 17428822

[B30] Ponikowski P. Voors A. A. Anker S. D. Bueno H. Cleland J. G. Coats A. J. (2016). ESC Guidelines for the diagnosis and treatment of acute and chronic heart failure: The Task Force for the diagnosis and treatment of acute and chronic heart failure of the European Society of Cardiology (ESC). Developed with the special contribution of the Heart Failure Association (HFA) of the ESC. Eur. J. Heart Fail. 18 (8), 2129. 10.1002/ejhf.592 27207191

[B31] Roger V. L. Go A. S. Lloyd-Jones D. M. Benjamin E. J. Berry J. D. Borden W. B. (2012). Heart Disease and Stroke Statistics--2012 Update: a Report from the American Heart Association. Circulation 125 (1), e2–e220. 10.1161/CIR.0b013e31823ac046 22179539 PMC4440543

[B32] Sarnoff S. J. Braunwald E. Welch G. H. Case R. B. Stainsby W. N. Macruz R. (1958). Hemodynamic Determinants of Oxygen Consumption of the Heart with Special Reference to the Tension-Time index. [J]. Am. J. Physiol. 192 (1), 148. 10.1152/ajplegacy.1957.192.1.148 13498167

[B33] Shimizu G. Hirota Y. Kita Y. Kawamura K. Saito T. Gaasch W. H. (1991). Left Ventricular Midwall Mechanics in Systemic Arterial Hypertension. Myocardial Function is Depressed in Pressure-Overload Hypertrophy. [J]. Circ. 83 (5), 1676–1684. 10.1161/01.CIR.83.5.1676 1827056

[B34] Taegtmeyer H. Young M. E. Lopaschuk G. D. Abel E. D. Brunengraber H. Darley-Usmar V. (2016). Assessing Cardiac Metabolism: A Scientific Statement from the American Heart Association. Circ. Res. 118 (10), 1659–1701. 10.1161/RES.0000000000000097 27012580 PMC5130157

[B35] Toh N. Ishii K. Kihara H. Iwakura K. Watanabe H. Yoshikawa J. (2016). Effect of ARB/Diuretics on Diastolic Function in Patients with Hypertension 2 (EDEN2) Trial Investigators. Effect of Diuretic or Calcium-Channel Blocker Plus Angiotensin-Receptor Blocker on Diastolic Function in Hypertensive Patients. Circ. J. 80 (2), 426–434. Epub 2015 Dec 25. PMID: 26725762. 10.1253/circj.CJ-15-0815 26725762

[B36] World Medical Association (2013). World Medical Association Declaration of Helsinki: Ethical Principles for Medical Research Involving Human Subjects. JAMA 310 (20), 2191–2194. 10.1001/jama.2013.281053 24141714

